# Point-of-care Ultrasonography of a Rare Cause of Hemoperitoneum

**DOI:** 10.5811/cpcem.2018.7.38210

**Published:** 2018-09-05

**Authors:** Kyle R. Kelson, Matthew Riscinti, Michael Secko, Ian S. deSouza

**Affiliations:** * SUNY Downstate Medical Center, Department of Emergency Medicine, Brooklyn, New York; † Stony Brook University Medical Center, Department of Emergency Medicine, Stony Brook, New York

## Abstract

A young woman presented to the emergency department with lethargy, hemodynamic instability, and diffuse abdominal tenderness. On point-of-care ultrasound (PoCUS), she was found to have intraperitoneal free fluid and a large pelvic mass, which were discovered intraoperatively to be hemoperitoneum due to ruptured vessels of a uterine leiomyoma. Although rare, a life-threatening, ruptured leiomyoma may be treated surgically if recognized in an expedient fashion. A PoCUS can aid the emergency clinician in prompt diagnosis.

## INTRODUCTION

While uterine leiomyomas are common among reproductive-aged women, they rarely result in acute life-threatening events. However, the vessels overlying a leiomyoma may rupture leading to hemoperitoneum.^1^,^3^–^6^,^8^–^10^ Patients with ruptured leiomyoma are often considered too unstable for confirmatory imaging and taken to the operating room without delay.^1^–^8^ Although uncommon, this high-acuity condition is worth considering in the hemodynamically unstable, young female patient due to its amenability to surgical intervention. This report describes a case of ruptured leiomyoma that was promptly diagnosed with point-of-care ultrasonography (PoCUS) in the emergency department (ED) and discusses pathophysiology, diagnosis, and management from the emergency medicine perspective.

## CASE REPORT

A 43-year-old, previously known to be healthy woman was brought to the ED by emergency medical services (EMS) after her husband found her to be confused. EMS provided oxygen by facemask and obtained peripheral venous access. Upon arrival to the ED, the patient demonstrated depressed mental status but when aroused, she complained of generalized weakness. Her heart rate was 80 beats per minute, blood pressure was 78/52 millimeters of mercury, respiratory rate was 14 respirations per minute, and temperature was 37.1° Celsius (98.8° Fahrenheit). The bedside glucose level was 170 grams per deciliter (g/dL). A cardiorespiratory monitor was attached to the patient, and one liter (L) crystalloid was infused with a pressure bag. The patient was somnolent but arousable, diaphoretic, and pale. Upon auscultation, the chest was clear and cardiac rhythm regular. Radial pulses were diminished in both upper extremities. There was mild, diffuse abdominal tenderness and a firm mass appreciated in the pelvis. Rectal examination revealed brown stool.

**Image 1:**
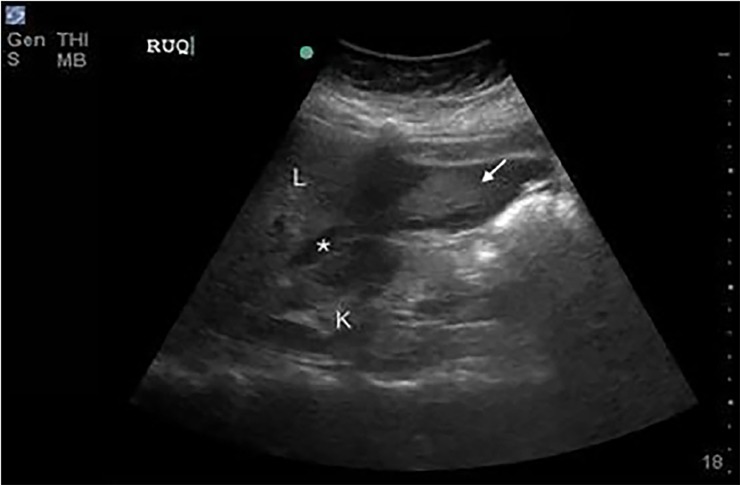
A right upper-quadrant image in a coronal plane with free fluid (*) noted in Morison’s pouch between the liver (L) and kidney (K). Hyperechoic (arrow) - material appreciated at liver tip is consistent with likely coagulated blood.

**Image 2:**
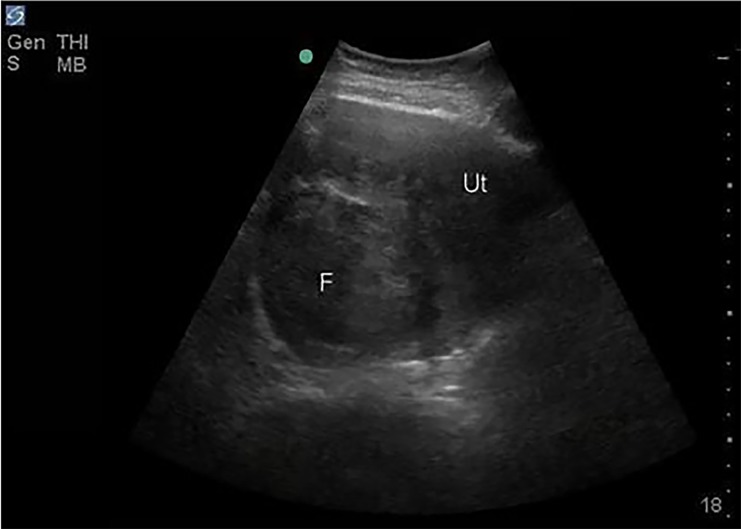
A trans-abdominal pelvic image in a transverse plane with a large fibroid (F) noted within the uterus (Ut).

The emergency physician performed point-of-care ultrasonography (PoCUS), specifically a Rapid Ultrasound for Shock and Hypotension (RUSH) examination. The PoCUS demonstrated a moderate amount of free fluid (Image 1) and a well-circumscribed mass adjacent to the uterus (Image 2). Laboratory analysis was significant for a venous lactate level 2.5 millimoles per liter (mmol/L), white blood cell count 11.46 cubic milliliter (K/uL), hemoglobin 9.0 g/dL, and negative urine human chorionic gonadotropin. The patient’s unstable condition precluded confirmatory computed tomography, and gynecology and general surgery were notified immediately for operative management of suspected hemoperitoneum. A repeat RUSH demonstrated an increased amount of free intraperitoneal fluid. While blood products were prepared, two additional L of crystalloid were infused.

Two hours after arrival, the urine output totaled 40 milliliters (mL), and the repeat hemoglobin fell to 5.0 g/dL. The gynecology consultant performed a bedside transvaginal sonogram and suspected the mass to be a uterine leiomyoma. The ED staff transfused the patient with three units of packed red blood cells, and collaborative gynecology and general surgery services brought the patient to the operating room (OR) for explorative laparotomy. The gynecology team noted a leiomyomatous uterus and 2.5 L of blood in the peritoneum. The source of hemorrhage was localized to multiple bleeding vessels overlying a leiomyoma, and the gynecology team performed a myomectomy. The OR staff transfused an additional five units of packed red blood cells and three units of fresh frozen plasma. The patient had an uneventful postoperative course and hospital stay. She was discharged from the hospital several days later and appeared healthy at postoperative checkups.

## DISCUSSION

Although uterine leiomyomas are common among reproductive-aged women, spontaneous life-threatening bleeding is exceedingly rare. Most reported cases seem to occur without insult,^3^–^7^,^10^ although one occurred after a large bowel movement,^1^ and another involved a laceration to the leiomyoma after a “vigorous coital experience.”^2^ There has also been report of bleeding that may have been exacerbated by pregnancy or delivery.^8^,^9^ These cases are consistent with the theory that increased intra-abdominal pressure may increase the risk of rupture of overlying vessels.^3^,^4^ The clinical presentation typically involves sudden hemodynamic collapse with abdominal pain, tenderness, or distention. Patients are uniformly found to have free intraperitoneal fluid on abdominal^4^–^8^ or pelvic ultrasonography,^1^,^3^ frequently with visualization of the leiomyoma.^1^,^3^,^5^–^7^

**CPC-EM Capsule**

What do we already know about this clinical entity?

Uterine leiomyoma are common among reproductive-age women and typically presents with pelvic pain or non-life threatening vaginal bleeding

What makes this presentation of disease reportable?

Rarely, uterine leiomyoma may rupture and present with hemoperitoneum, a potentially life-threatening condition

What is the major learning point?

A female patient who presents with hemodynamic instability and abdominal pain, tenderness, or distention may be promptly diagnosed with ruptured uterine leiomyoma using point-of-care ultrasonography (PoCUS)

How might this improve emergency medicine practice?

Prompt PoCUS diagnosis of ruptured uterine leiomyoma may expedite urgent resuscitation with blood products and transfer to the operating room for definitive intervention

The most frequent cause of hemoperitoneum related to uterine leiomyoma is the rupture of an overlying, superficial artery or vein.^1^,^3^–^10^ However, there has been report of spontaneous bleeding from leiomyoma itself.^7^ From the emergency medicine perspective, the management approach should be as for any unstable patient with signs of peritonitis and intraperitoneal free fluid on RUSH – resuscitation with blood products and consultation with appropriate surgical consulting services. The definitive treatment may include ligation of the bleeding vessel,^5^,^8^ myomectomy,^1^–^4^,^6^ or hysterectomy,^7^,^9^,^10^ depending on origin of bleeding and age and reproductive status of the woman.

## CONCLUSION

Despite uterine leiomyoma being a common tumor in young women, the rupture of associated blood vessels resulting in hemoperitoneum appears to be exceptionally rare. However, this condition may be fatal, and swift action is required for diagnosis and surgical intervention. This case report is unique in that it presents sonographic images to demonstrate the utility of PoCUS for the diagnosis of ruptured uterine leiomyoma. This diagnosis should be considered in any female patient who presents with hemodynamic instability and abdominal pain, tenderness, or distention. A quick diagnosis with PoCUS may expedite urgent resuscitation with blood products and transfer to the OR for definitive intervention.

Documented patient informed consent and/or Institutional Review Board approval has been obtained and filed for publication of this case report.
